# CRTC2 promotes paclitaxel resistance by inducing autophagy in ovarian cancer in part via the PI3K-AKT signaling axis

**DOI:** 10.7150/jca.82233

**Published:** 2023-04-09

**Authors:** Chaoyang Ou, Chen Peng, Yilang Wang, Shiyu Lu, Xinli Yu, Qian He, Aiqin He, Li Zhang

**Affiliations:** 1 Department of Cancer Research Center, Nantong Tumor Hospital, The Affiliated Tumor Hospital of Nantong University, Nantong, China; 2 Department of Gynecology Oncology, Nantong Tumor Hospital, The Affiliated Tumor Hospital of Nantong University, Nantong, China; 3 Department of Gynecology and Obstetrics, The Affiliated Hospital of Nantong University, Nantong, China; 4 Department of Oncology, The Affiliated Maternity and Child Health Care Hospital of Nantong University, Nantong, China

**Keywords:** ovarian cancer, paclitaxel resistance, CRTC2, PI3K-AKT, autophagy

## Abstract

**Background:** Ovarian cancer is the most malignant gynecological disease, which seriously threatens female physical and mental health. Paclitaxel is a first-line chemotherapy drug in the clinical treatment of ovarian cancer, but drug resistance has become an important factor affecting the survival of ovarian cancer patients. However, the main mechanism of chemotherapy resistance in ovarian cancer remains unclear. In this study, we analyzed the Integrated Gene Expression Database (GEO) dataset using comprehensive bioinformatics tools to provide new therapeutic strategies and search for prognostic targets for ovarian cancer.

**Methods:** Ovarian cancer related genes were extracted from GSE18520 by bioinformatics method. Differentially expressed genes (DEGs) were obtained by differential analysis, and related genes and functions were elucidated. The key gene CRTC2 was identified by prognostic analysis. Immunohistochemistry was used to detect the expression of CRTC2 in chemotherapy-resistant and chemotherapy-sensitive ovarian cancer tissues. Functional analysis (cell assay) confirmed the role of CRTC2 in paclitaxel resistance. Autophagy related proteins were detected by Western blot. Autophagy flux analysis was performed using the GFP/RFP-LC3 adenovirus reporter.

**Results:** A total of 3,852 DEGs were identified in the GEO microarray dataset. Key genes were screened by prognostic analysis. We found that CRTC2 was highly expressed in chemoresistant tissues of ovarian cancer. In 110 patients with ovarian cancer, high expression of CRTC2 was associated with poorer prognostic factors and shorter survival. At the same time, we found that CRTC2 can promote the proliferation and invasion ability of ovarian cancer cells. In addition, CRTC2 can affect the expression of PI3K, AKT, autophagic flux and sensitivity to paclitaxel chemotherapy in ovarian cancer.

**Conclusion:** CRTC2 can affect autophagy partially through PI3K-AKT signaling pathway, and then affect the sensitivity of ovarian cancer to paclitaxel chemotherapy. CRTC2 may be a potential predictor or target for ovarian cancer therapy.

## Introduction

Ovarian cancer is one of the common malignant tumors of the female reproductive system, second only to breast cancer and cervical cancer, but the highest degree of malignancy of ovarian cancer is the main cause of death of gynecological cancer 1. Approximately 70% of patients with ovarian cancer are diagnosed in advanced stages due to the lack of specific clinical manifestations in the early stages 2-3. At present, the standard treatment plan for ovarian cancer is active surgery to reduce tumor, and then platinum plus paclitaxel-based chemotherapy. Despite recent advances in surgical treatment, chemotherapy and targeted therapy, the clinical outcomes among patients are still not optimistic, and the main reason is the development of chemotherapy resistance 4-5. At present, there are no clinically reliable biomarkers that can predict the response to chemotherapy in ovarian cancer, and the underlying mechanisms of chemotherapy resistance are still deficient. Therefore, it is of great significance to continue to explore the potential mechanism of chemotherapy resistance in ovarian cancer and find new strategies to reverse chemotherapy resistance in ovarian cancer.

CRTC2 belongs to the CREB-regulated transcription coactivator (CRTC) family. The CRTC family consists of three members: CRTC1, CRTC2 and CRTC3, which are expressed in several tumors 6-7. CRTC2 is a transcriptional activator of CREB and enhances CREB transcriptional activity by binding to the leucine zipper DNA binding region of CREB. CRTC2 and its downstream targets can mediate and regulate estrogen receptor-aromatase pathway, CAMP and other metabolic signaling pathways 8-9. CRTC2 is involved in the occurrence and development of various tumors. Some studies have shown that CRTC2 promotes the migration and invasion of non-small cells 10. In liver kinase B1 (LKB1) mutant non-small cell lung cancer, CRCT2 is constitutively activated by SIKs inactivation and promotes the progression of NSCLC by binding to CREB to initiate transcription of downstream genes 11. It has also been found that overexpression of CRTC2 can promote the proliferation of colorectal cancer 12. CRTC2 can also be used as a novel prognostic biomarker for pathological outcome and biochemical recurrence after radical prostatectomy in patients with prostate cancer 13. Carper, MB, et al. confirmed that activation of MEKK1-p38 signal axis can make CRTC2 locate in the nucleus, increase the transcription activity of CRTC2/CREB, and thus enhance the progress of HNSCC 14. However, the role of CRTC2 in ovarian cancer remains unclear.

Autophagy is a highly evolutionary and conservative process in all eukaryotes, which can lead to the degradation of cytoplasm in a lysosomal dependent manner. The degradation of autophagy recovers the cellular components and provides the energy needed to maintain homeostasis during metabolic stress 15. However, the effect of autophagy on cancer cells is two-side 16. Autophagy can protect the genome from damage and inhibit the occurrence of tumors, but this process may also activate metabolic stress response to promote tumor growth 17-18. Seok, S et al. have proved that CREB can up regulate autophagy genes by recruiting the coactivator CRTC2, including Atg7, Ulk1 and Tfeb. The FXR-CREB/CRTC2 axis is identified as the key physiological switch for regulating autophagy, so as to continuously regulate autophagy in the feeding/fasting cycle 19. Some studies have revealed the influence of autophagy on tumor drug resistance 20. Paclitaxel, as a common chemotherapy drug, may lead to autophagy to promote drug resistance 21. However, to our knowledge, the role of CRTC2 in inducing autophagy and paclitaxel resistance in tumors including ovarian cancer has not been reported so far.

In this study, we demonstrated for the first time that CRTC2 is overexpressed in ovarian cancer and promotes ovarian cancer proliferation and invasion, and demonstrated that its overexpression is significantly associated with poor patient survival. At the same time, in addition, we found that paclitaxel induced up-regulation of CRTC2 expression and autophagy response, thereby providing paclitaxel cytoprotection. Therefore, our findings suggest that CRTC2 plays a key role in paclitaxel resistance by inducing autophagy. CRTC2 may be a potential predictor or target for ovarian cancer therapy.

## Materials and Methods

### Microarray data acquisition and identification of DEGs

We obtained the gene chip dataset GSE18520 related to ovarian cancer through the GEO module of the National Center for Biotechnology Information (NCBI), and downloaded the relevant microarray data. The platform of GSE18520 is GPL570, [HG-U133_Plus_2] Affymetrix Human Genome U133 Plus 2.0 Array, which includes 10 normal ovarian samples and 53 ovarian tumors.

All genes in normal and cancer samples were analyzed through online website GEO2R (https://www.ncbi.nlm.nih.gov/geo/geo2r/?acc=GSE18520) to identify DEGs and then download corresponding files for subsequent analysis.

### Functional enrichment

The potential functions of DEGs were analyzed by functional enrichment. Gene ontology (GO) is a widely used tool to annotate functional genes, especially molecular functions (MF), biological processes (BP) and cellular components (CC). Kyoto Encyclopedia of Genes and Genomes (KEGG) enrichment analysis is a practical method for analyzing gene function and related high-level genomic function information. In order to better understand the carcinogenesis of the target gene, the ClusterProfiler (version 3.18.0) in the R software package is used for functional enrichment. P < 0.05 was considered to be statistically significant.

### Acquisition of eligible genes

Keywords of “PI3K-AKT” and “AMPK” were input in the online website GeneCards (https://www.genecards.org/) to download the appropriate gene sets, thus obtaining the eligible genes. Then, the DEGs and two gene sets were applied to plot the overlapping gene Venn diagrams employing the online website devoted to manufacturing Venn diagrams (http://bioinformatics.psb.ugent.be/webtools/Venn/).

### Survival analysis

The Kaplan-Meier plotter (https://kmplot.com/analysis/index.php?p=background) is a serviceable website tool, which contains considerable information on several cancers, including ovarian cancer. Survival analysis was conducted using the Kaplan-Meier plotter, and the log-rank p value and hazard ratio (HR) with 95% confidence intervals were computed and shown on the plot.

### Single-sample gene set enrichment analysis

Spearman correlation analysis between single gene and pathway score was conducted through R software GSVA package (version 4.0.3). p < 0.05 was considered to be statistically significant.

### Immunohistochemical

The specimens were stained by immunohistochemistry (SP). The thickness of the sections was about 4μm. The slices were baked in an oven at 85℃ for 30min, dewaxed with xylene and dehydrated with ethanol. The alkaline repair solution was heated at high pressure for antigen repair, cooled to room temperature, blocked with peroxidase blocker at room temperature for 20min, and the primary antibody CRTC2 (ab244419, Abcam, UK) was used at 4℃ overnight (dilution concentration: 1:50). The next day, the cells were rewarmed to room temperature and incubated with secondary antibody (PV-6001, Zhongshan Biotech, Beijing, China) for 20min at room temperature, and rinsed with PBS buffer for 5min/3times between each step. DAB (Dako, DK) for 2min, hematoxylin for 30s, ethanol dehydration, xylene dewaxing, and neutral gum seal. The degree of staining was divided into unstained, light yellow, yellow, brown or dark brown, and the corresponding staining intensity was -, +, ++ and +++, the score was 0, 1, 2 and 3, respectively. The positive areas were < 20%, 21%-50%, 51-75% and > 75%, and the scores were 1, 2, 3 and 4, respectively. The composite score is the product of two indicators.

### Cell culture and transfection

The normal ovarian epithelial cell line IOSE80, and ovarian cancer cell lines HO8910PM, SKOV3, CAOV3, and A2780 were purchased from Meisen Chinese Tissue Culture Collections. The cells were cultured in RPMI 1640 (HyClone, USA) medium with 10% fetal bovine serum (FBS, Gibco, USA) and 1% penicillin-streptomycin (NCM Biotech, China) solution in a 5% CO_2_ incubator at 37°C. Paclitaxel was purchased from selleck.

The cells of logarithmic growth phase were inoculated in the 6-well plate with 70%-80% density. This study verified the role of CRTC2 in ovarian cancer by transfecting shNC, shCRTC2, over expression CRTC2 (OE-CRTC2), and GFP/RFP-LC3 adenovirus (Jikai Gene, Shanghai, China), and operated experiments according to the instructions of RNA transfection kit (Thermo Fisher Scientific).

### Selection of patient specimen

A total of 110 formalin-fixed and paraffin-embedded tissue samples were collected randomly from patients diagnosed with ovarian serous, mucinous, endometrioid, and clear cell carcinoma between January 2013 and January 2016 from the Affiliated Tumor Hospital of Nantong University for immunohistochemical analysis. The specific inclusion criteria are as follows: 1. All patients were treated with primary postoperative paclitaxel chemotherapy. 2. The clinical characteristics of patients are complete, including age, pausimenia, distant metastasis, neoadjuvant chemotherapy, ascites, FIGO stage, lymphatic metastasis and chemosensitivity. 3. The patients had whole follow-up data, and the follow-up deadline is July 31, 2019. 4. There was no complications or secondary malignant tumor. All pathological diagnoses were reconfirmed by blind examination by pathologists. This study was approved by the Ethics Committee of the Affiliated Tumor Hospital of Nantong University.

### Western blotting

Total protein was isolated from cells using RIPA (NCM Biotech, China) containing PMSF (Beyotime, China). The extracted proteins were separated on 10-15% SDS PAGE gels, transferred to PVDF membranes, blocked with 5% milk, and incubated with primary antibodies overnight at 4°C. After incubation with secondary antibodies, proteins can be visualized with ECL (Tanon, China) luminescence solution. The primary antibodies included rabbit anti-CRTC2 (ab244419, Abcam, UK), anti-BECN1 (ab62557, Abcam, UK), anti-LC3 (ab51520, Abcam, UK), anti-PI3K (Santa Cruz, USA), anti-AKT (Santa Cruz, USA), and anti-p-AKT (Santa Cruz, USA). Mouse anti-GAPDH (Proteintech, China) was used as a normalized control.

### Cell viability detection

24h after transfection of A2780 and SKOV3 cells with shCRTC2 and OE-CRTC2, per well cells of 2,000 were inoculated in the 96-well plates. Cells were exposed to different final concentrations of paclitaxel (0, 2, 5, 10, 20, 50nM; or 0, 5, 10, 20, 50, 100nM) for 24h. Each concentration was repeated in triplicate wells. Then, media were exchanged according to the manufacturer's instructions, and cell viability was evaluated after 24h with cell counting Kit-8 (CCK-8, Vazyme, China). The cell viability was assessed by detecting OD450 with an automatic spectrophotometer.

### Clonogenic assay

The transfected A2780 and SKOV3 cells were inoculated into 6-cell plates (500 cells/well) and allowed to grow for 14 days until colonies were visible. The cells were fixed by 4% paraformaldehyde solution (Sangon Biotech, China), and stained with crystal violet (Sangon Biotech, China). Finally, the images were provided.

### Transwell

The invasion ability of SKOV3 and A2780 cells was detected by transwell chamber containing 8μm pore size polycarbonate filter. Briefly, 500μl of complete medium was added to a 24-well plate. The equivalent of 5×10^4^ cells were seeded in the culture chamber and incubated with 200μl medium at 37°C in 5% CO_2_. After 48h of incubation, the invading cells opposite the filter were gently rinsed with PBS, fixed with 4% paraformaldehyde solution, and stained with 0.5% crystal violet. After complete rinsing with PBS, cells were photographed using a microscope.

### Autophagic flux analysis

The shCRTC2 and OE-CRTC2 transformed A2780 cells were transferred to 24-well plates with cover glass and then transfected with GFP/RFP-LC3 adenovirus at a multiplicity of infection of 50 for 24h. The transfected cells were first cleaned with PBS for three times, then fixed with 4% paraformaldehyde solution, and then scanned under a confocal microscope (Leica, German). Randomly select 5 regions in each group of samples to calculate the number of GFP and RFP puncta.

### Statistical analyses

SPSS 25.0 statistical software was used to analyze the relationship between the expression of CRTC2, BECN1 and clinical pathological characteristics using chi square test. Kaplan Meier survival curve was used to analyze the relationship between the expression of CRTC2 and prognosis of ovarian cancer patients. p<0.05 was statistically significant.

## Results

### Identification of DEGs and functional enrichment analysis

**Figure 1:**
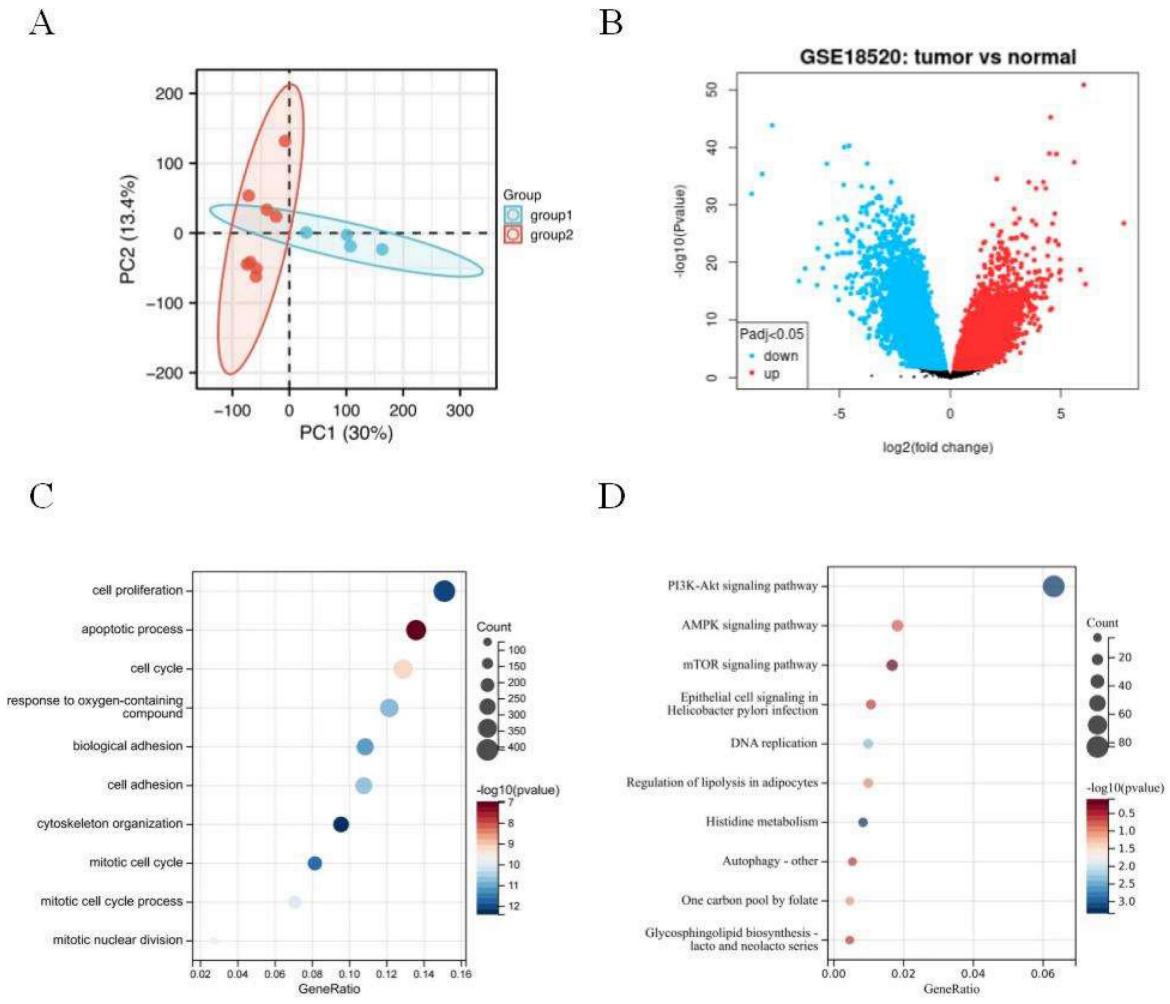
**Identification of DEGs in GSE18520 and functional enrichment analysis.** (A) Principal component analysis for GSE18520. Group 1 represents normal samples, Group 2 represents ovarian cancers. (B) Volcano plot of the 3,852 DEGs. The red dots represent the significantly up-regulated genes, and the blue dots the significantly down-regulated genes. (C) GO analysis of DEGs. (D) KEGG analysis of DEGs.

The principal component analysis (PCA) was employed to estimate the regression of the data within the group, confirming the favorable repeatability of the data in GSE18520 (Figure 1A). The gene expression data between 10 normal ovarian samples and 53 ovarian tumors in GSE18520 was analyzed by GEO2R online website. A total of 3,852 DEGs were identified, which were showed by volcanic map (Figure 1B). Then, 3,852 DEGs analyzed by GO and KEGG enrichment. GO analysis revealed that the DEGs were mainly related to the cell proliferation, apoptotic process, and cell cycle (Figure 1C). KEGG enrichment showed that the DEGs were mainly related to PI3K-AKT, AMPK, and mTOR signaling pathways (Figure 1D).

### Identification and analysis of key gene CRTC2

**Figure 2:**
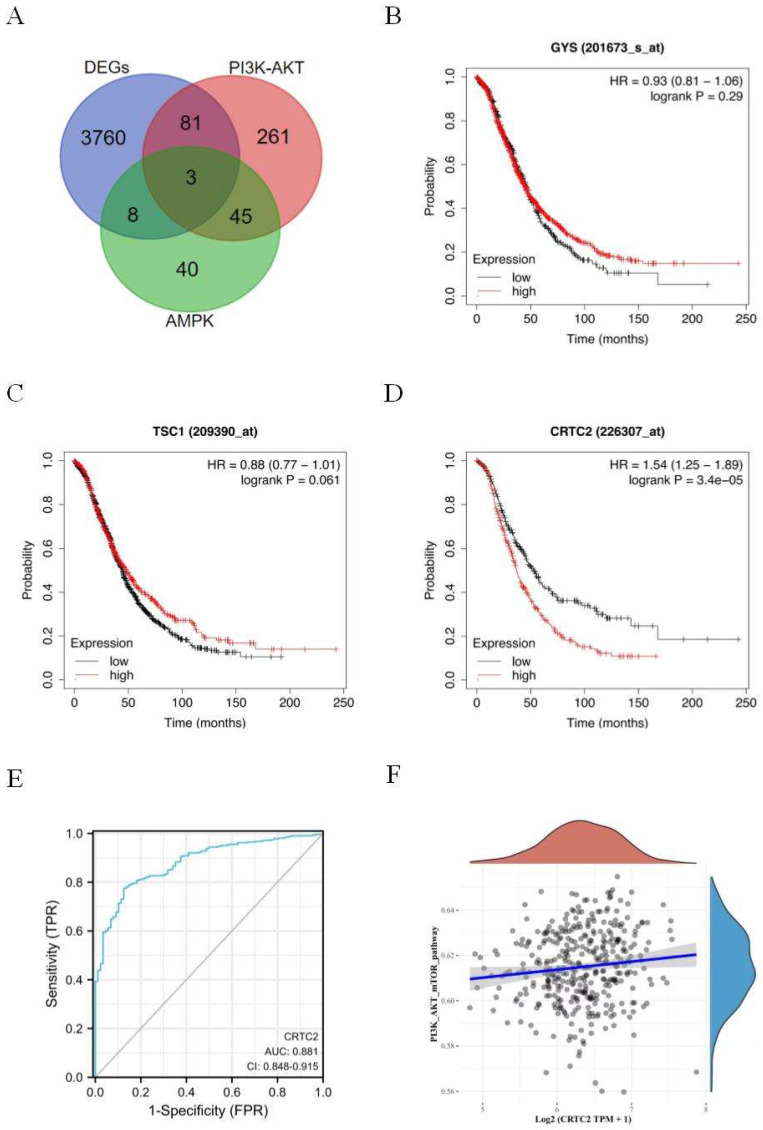
** Identification and analysis of CRTC2.** (A) The Venn diagram of DEGs, PI3K-AKT genes, and AMPK-related genes. (B-D) Kaplan-Meier analysis was used to determine the prognostic role of GYS, TSC1, and CRTC2 in ovarian cancer. (E) The ROC curve of TCGA-OV according to the expression of CRTC2. p<0.001. (F) The PI3K-AKT-mTOR signal pathway positively correlated with the high expression of CRTC2**.** p=0.009.

Signaling pathways of PI3K-AKT and AMPK are closely related to the occurrence, development and drug resistance of tumors. In order to further explore the effect mechanism of signaling pathways on ovarian cancer. Three genes GYS, TSC1, and CRTC2 were obtained by Venn diagram of DEGs, PI3K-AKT, and AMPK genes (Figure 2A). Based on the Kaplan-Meier Plotter database, the prognostic potential of 3 genes in ovarian cancer was explored. The results showed that only the expression level of CRTC2 in ovarian cancer was negatively correlated with the overall survival of patients [HR=1.54 (1.25-1.89), p < 0.001] (Figure 2B-D). ROC curve was used to verify the prognostic ability of CRTC2 in TCGA-OV cohort. Figure 2E reveals the AUC of CRTC2 to be 0.881 (p < 0.001). Then, the RNAseq data of ovarian cancer and corresponding clinical information were obtained from the Cancer Genome Atlas (TCGA) database. Subsequently, we found that CRTC2 was positively correlated with PI3K-AKT-mTOR signaling pathway through the analysis of R software GSVA package (p = 0.009) (Figure 2F). Therefore, CRTC2 has high sensitivity and specificity and can be used as a candidate biomarker for the prognosis of ovarian cancer.

### Validation of the expression and prognostic value of CRTC2

**Figure 3:**
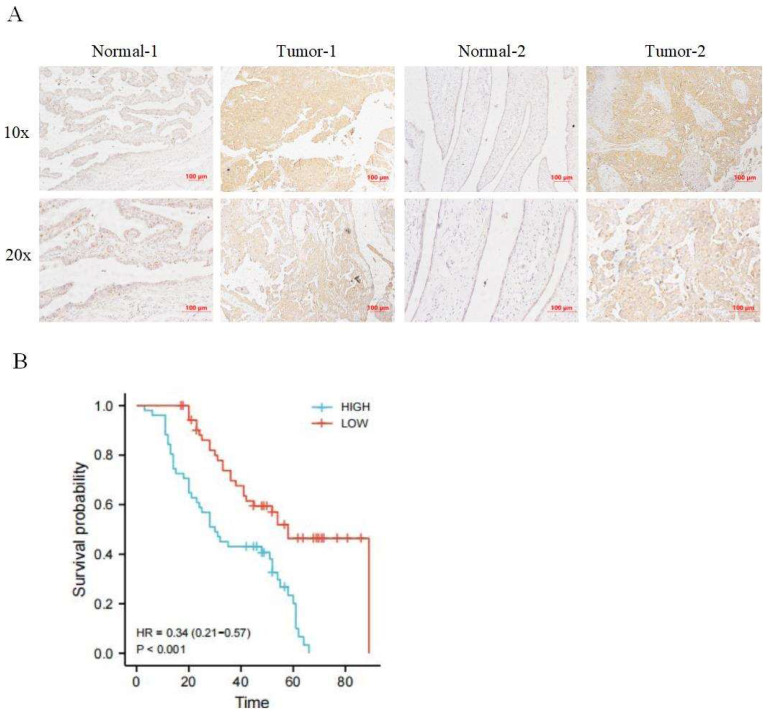
** Expression and prognosis of CRTC2 in ovarian cancer.** (A) The IHC showed that CRTC2 protein is highly expressed in tumor tissues. (B) Kaplan Meier curve analysis of patients with high and low expression levels of CRTC2.

Immunohistochemical staining was used to detect the expression of CRTC2 in 110 cases of ovarian cancer tissues and corresponding normal tissues in tissue microarray, and it was confirmed that the expression of CRTC2 in ovarian cancer tissues was higher than that in corresponding normal tissues (Figure 3A). According to the median method, 110 patients with ovarian cancer were divided into high expression group and low expression group of CRTC2. There were 52 patients with low expression of CRTC2 and 58 patients with high expression. As shown in Table 1, the highly expressed CRTC2 has a tendency of distant metastasis (p = 0.005) and higher FIGO stage (p = 0.018) of ovarian cancer. Kaplan-Meier analysis was used to demonstrate the relationship between CRTC2 expression and overall survival, confirming that ovarian cancer with high CRTC2 expression had a shorter overall survival, while ovarian cancer with low CRTC2 expression had a longer overall survival (p < 0.001) (Figure 3B).

### Carcinogenic effect of CRTC2 in ovarian cancer

**Figure 4:**
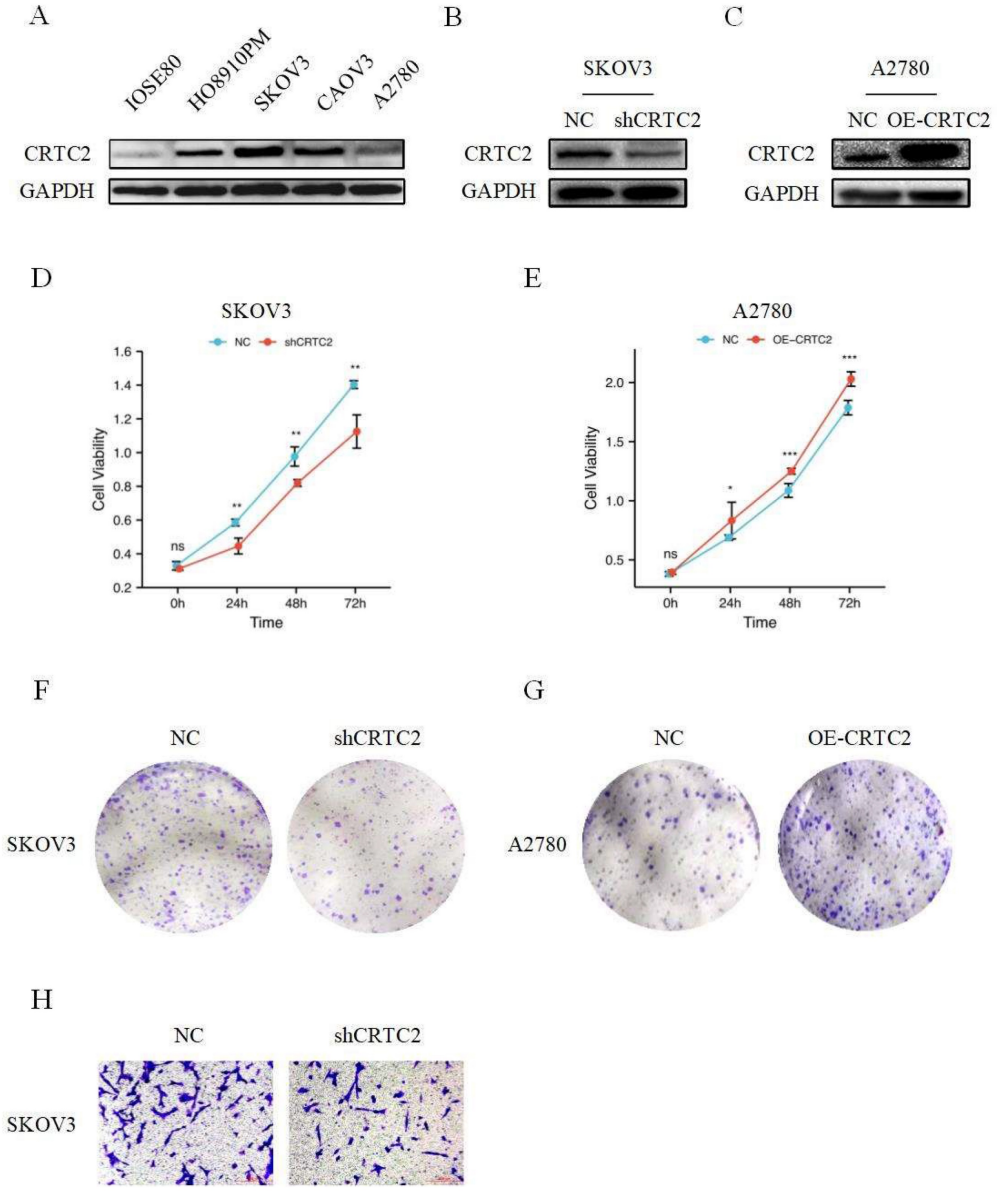
** CRTC2 promotes the development of ovarian cancer.** (A) The expression of CRTC2 in normal ovarian epithelial cell IOSE80, and in ovarian cancer cells HO8910PM, SKOV3, CAOV3, A2780. (B, C) Western blotting was used to verify the knockdown and overexpression efficiency of CRTC2. (D, E) CCK-8 assay was used to detect the proliferation ability of CRTC2 after knockdown and overexpression. (F, G) Colony forming assay. (H) Transwell assay was used to detect the invasive ability.

As shown in Figure 4A, CRTC2 expression was higher in cancer cells than in normal ovarian epithelial cells. To verify the role of CRTC2 in the development of ovarian cancer, we chose to knockdown CRTC2 in SKOV3 cells and overexpress CRTC2 in A2780 cells. Western blot analysis also confirmed the knockdown efficiency (Figure 4B) and overexpression efficiency of CRTC2 (Figure 4C). CCK-8 and clone formation assays were used to detect the effects of CRTC2 on cell proliferation and invasion. Specifically, CCK-8 assay showed that inhibition of CRTC2 decreased SKOV3 proliferative activity (Figure 4D), and overexpression of CRTC2 increased A2780 proliferative ability (Figure 4E). Clone formation assay revealed that knockdown of CRTC2 inhibited the colony count of SKOV3 cells (Figure 4F), and overexpression of CRTC2 increased the colony count of A2780 cells (Figure 4G). Through transwell assay, we found that the invasion ability of SKOV3 cells with silence of CRTC2 was decreased (Figure 4H). In conclusion, we found that CRTC2 gene can promote the development of ovarian cancer.

### CRTC2 reduces the efficacy of paclitaxel in ovarian cancer cells

**Figure 5:**
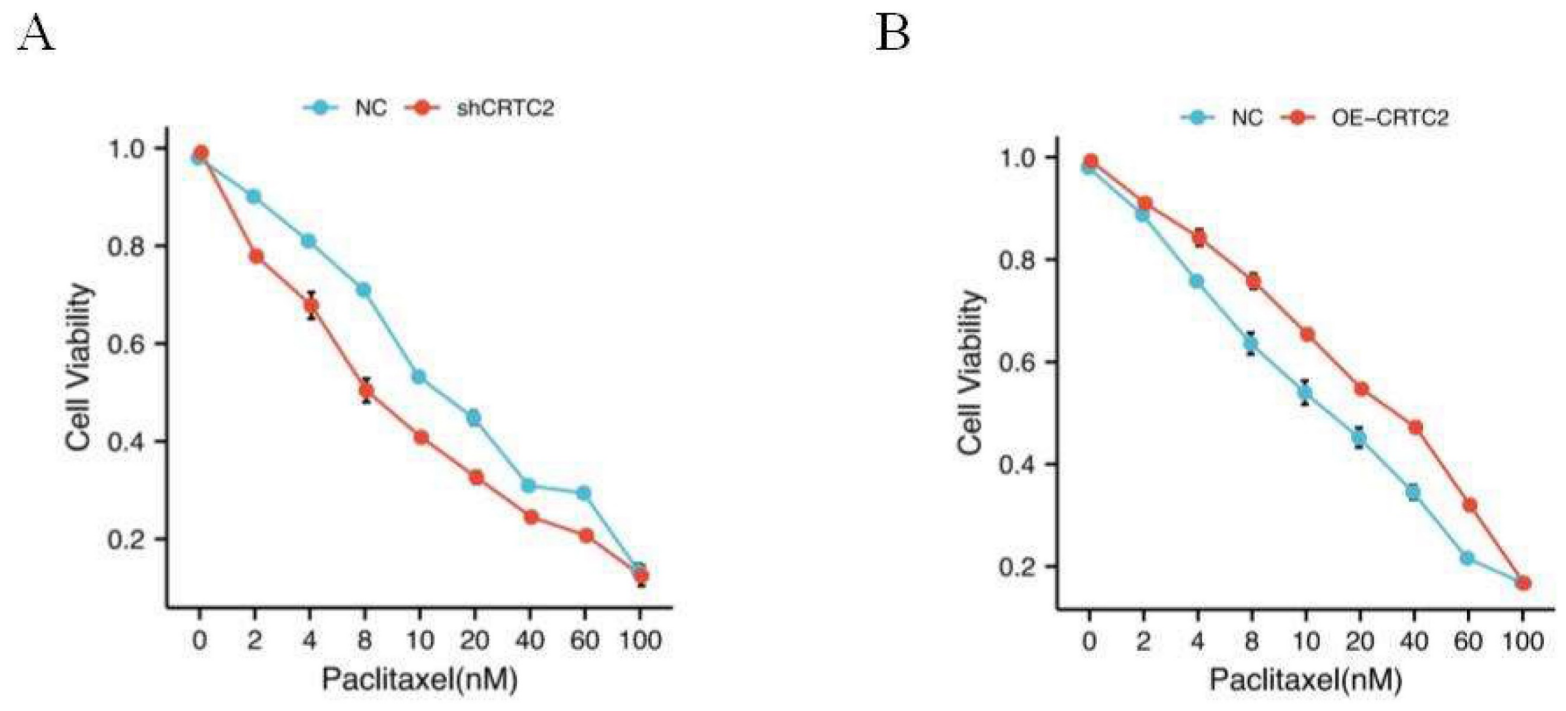
** CRTC2 modulates paclitaxel sensitivity**. (A) IC50 value of paclitaxel in SKOV3 cells with CRTC2 silence. p<0.05. (B) IC50 value of paclitaxel in A2780 cells with CRTC2 overexpression. p<0.05.

As shown in Table 1, in the tissues with high expression of CRTC2, the chemoresistance rate of ovarian cancer was 69.2% (45/65 cases), while in the tissues with low expression of CRTC2, the chemoresistance rate of ovarian cancer was only 44.4% (20/45 cases), and the difference was statistically significant (p = 0.009). In addition, high CRTC2 expression was significantly associated with neoadjuvant chemotherapy (p = 0.043) (Table 1). Based on this discovery, we tested the effect of paclitaxel, a first-line chemotherapy drug for ovarian cancer, on the knockdown and overexpression of CRTC2 in ovarian cancer cell lines. We found that the IC50 value of paclitaxel was decreased in SKOV3 cells with CRTC2 knockdown compared with control cells (Figure 5A). In contrast, A2780 cells overexpressing CRTC2 had higher IC50 values of paclitaxel, which protected ovarian cancer cells from paclitaxel damage (Figure 5B). These results indicate that the down-regulation of CRTC2 enhances the sensitivity of ovarian cancer cells to paclitaxel.

### Paclitaxel resistance due to up-regulation of CRTC2 is associated with autophagy

**Figure 6:**
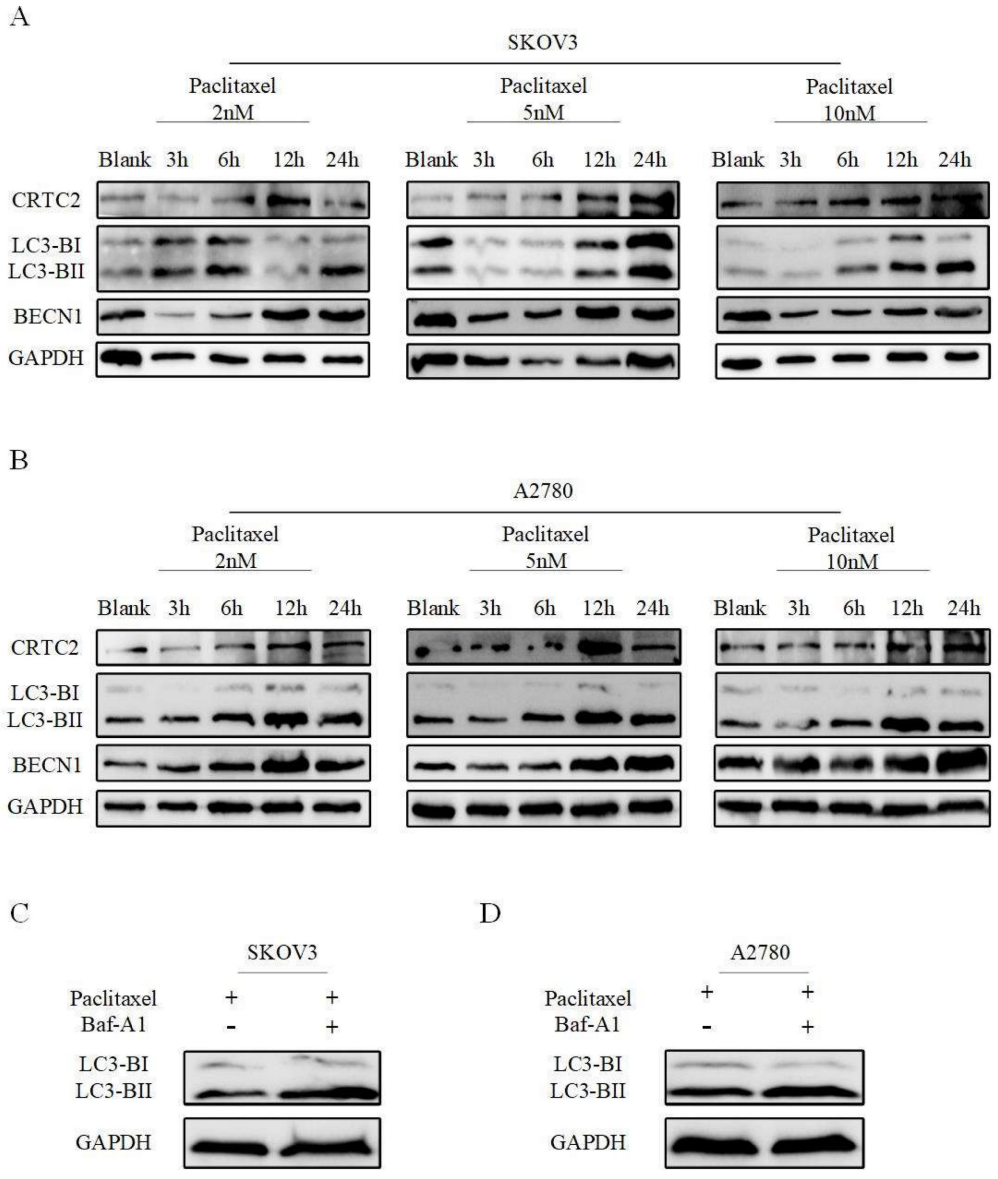
** Paclitaxel exposure upregulates the expression of CRTC2 and autophagic proteins.** SKOV3 (A), A2780 (B) cells were plated in 6-well plates overnight and reached 60% to 70% confluence when paclitaxel was added in the complete medium at final concentration 2, 5, and 10 nM. Total cell lysates were extracted after 3, 6, 12, and 24h of paclitaxel treatment. Protein levels of LC3B, BECN1, and CRTC2 were detected by western blotting. Cells without paclitaxel treatment were assigned as blank control (Blank). (C, D) SKOV3, A2780 cells treated with 10 nM paclitaxel with and without 10 nM Baf-A1. Total cell lysates were collected 48h after transfection. Protein levels of LC3B were determined by western blotting.

We previously found that CRTC2 is associated with PI3K-AKT-mTOR and AMPK signaling pathways. These pathways are closely related to autophagy. Next, we explored whether CRTC2 could affect the autophagy level in ovarian cancer cells. A2780 and SKOV3 cells were exposed to paclitaxel at different concentrations (2, 5 and 10 nM) and times (3, 6, 12 and 24h). The amount of CRTC2 protein was found to be increased after time increased exposures to paclitaxel at various concentrations in 2 cell lines. Moreover, paclitaxel also simultaneously induced upregulation of BECN1, and aggregation of the protein levels of the cleaved and lapidated form of MAP1LC3B-II/LC3B-II (microtubule-associated protein 1 light chain 3 β), which were observed along with CRTC2 upregulation (Fig 6A-B). Then, autophagic flux was also investigated by estimating LC3B-II transform by western blotting in the presence or absence of BafA1 (bafilomycin A_1_), a vacuolar HC/ATPase inhibitor that prevents lysosomal degradation. Moreover, LC3B-II was increased when SKOV3 and A2780 cells were exposed to paclitaxel and further accumulated after BafA_1_ treatment (Figure 6C-D). That's suggest an increase of autophagic flux after paclitaxel treatment in ovarian cancer cells. Therefore, to our findings, the reason of paclitaxel resistance in ovarian cancer may be the formation of increased CRTC2 and autophagy.

### CRTC2 plays a crucial role in the autophagy in ovarian cancer and partially due to the PI3K-AKT signaling pathway

**Figure 7:**
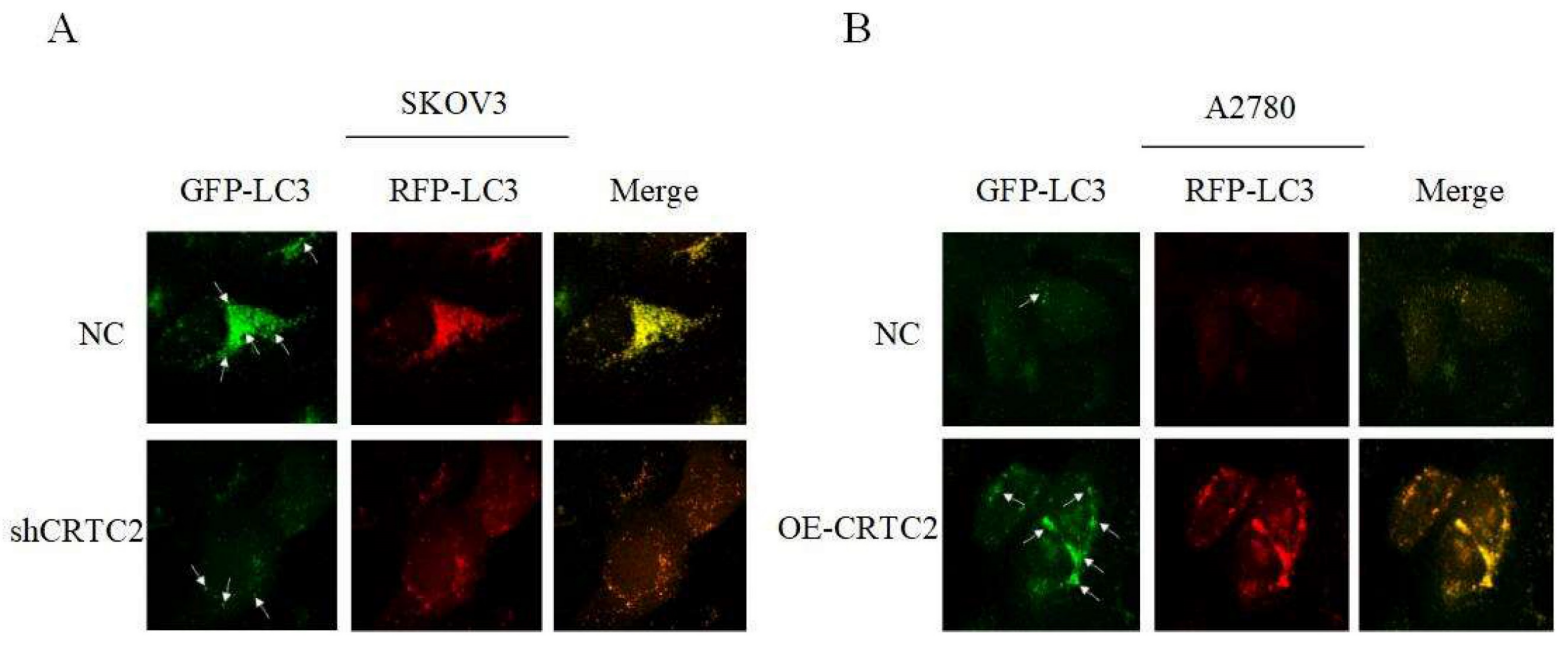
** Effect of CRTC2 expression on autophagy in ovarian cancer cells.** (A) SKOV3 cells with CRTC2 inhibition. (B) A2780 cells with CRTC2 overexpression.

Previously, we found that CRTC2 expression and autophagic flux increased in response to paclitaxel exposure. To demonstrate that CRTC2 is involved in the regulation of autophagy. We knocked down CRTC2 in SKOV3 cells by lentivirus, and overexpressed CRTC2 in A2780 cells. Subsequently, we detected autophagy flux using the GFP/RFP-LC3 adenovirus reporter. When autophagosomes fuse with lysosomes, GFP fluorescence is quenched, and only red fluorescence can be detected. It indicated that autophagosomes were not bound to lysosomes when GFP and RFP fluorescence appeared simultaneously. As shown in Figure 7A, yellow puncta (GFP/RFP-LC3) were reduced in SKOV3 cells after CRTC2 knockdown compared with control cells. However, the yellow puncta (GFP/RFP-LC3) increased in A2780 cells overexpressing CRTC2 (Figure 7B). Therefore, CRTC2 affects the fusion of autophagosomes and lysosomes.

**Figure 8:**
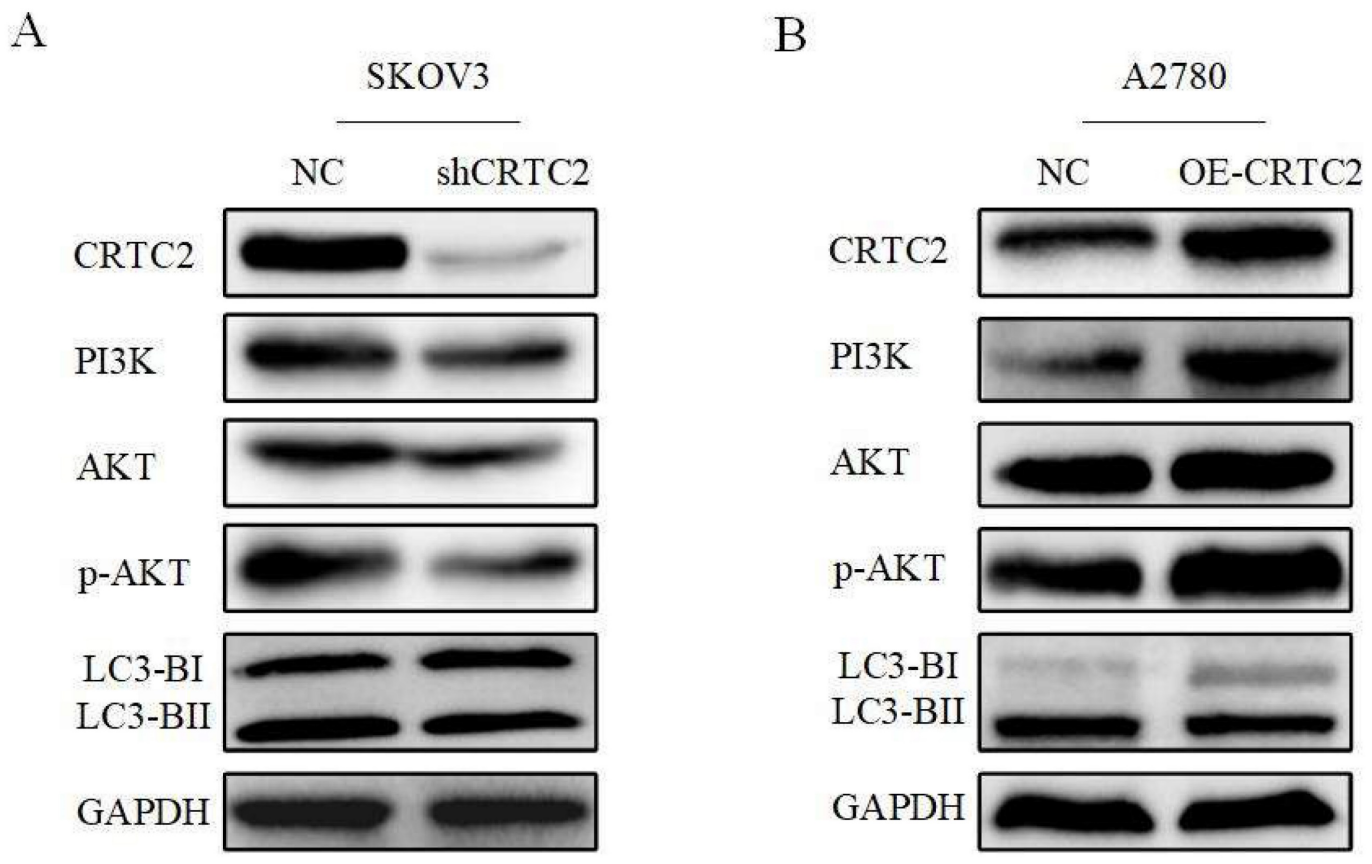
** CRTC2 induces autophagy through PI3K-AKT signaling pathway and affects the expression of autophagy markers.** (A) The expression of PI3K, AKT, and p-AKT and autophagy markers in SKOV3 cells with CRTC2 knockdown. (B) The expression of PI3K, AKT, and p-AKT and autophagy markers in A2780 cells with CRTC2 overexpressed.

Based on the above-described results, we confirmed that CRTC2 played a vital role in the autophagy to affect the chemoresistance of ovarian cancer cells. Previously, we found that CRTC2 was associated with PI3K-AKT signaling pathway through the database. We hypothesized that the regulatory effect of CRTC2 on ovarian cancer autophagy is partly attributed to PI3K-AKT signaling pathway. Based on these assumptions, we aimed to detect relevant effectors in this pathway by western blotting. As shown in Figure 8A, in SKOV3 cells with silence of CRTC2, the expression of PI3K was decreased, the expression p-AKT was decreased but not AKT, and the ratio of LC3-II/LC3-I was decreased. In A2780 cells overexpressing CRTC2, the expression of PI3K, p-AKT and LC3-II/LC3-I increased (Figure 8B). To sum up, CRTC2 plays a crucial role in the autophagy in ovarian cancer and partially due to the PI3K-AKT signaling pathway.

## Discussion

Ovarian cancer has the second highest mortality rate and the fourth highest incidence rate among gynecological malignancies 22. Although treatment advances and outcomes for ovarian cancer have improved over the past few decades, the poor overall survival rate of ovarian cancer poses a serious threat to women's physical and mental health 23. The combination of chemotherapy and surgery is considered to be the main treatment for ovarian cancer, but the overall five-year survival rate of patients with ovarian cancer has not improved significantly 24. The main reason is that most patients relapse within 2-3 years. With the increase of chemotherapy cycle, the recurrence interval of patients is shortened, the dose limiting toxicity of chemotherapy drugs increases, and finally the sensitivity of patients to drugs decreases 25-26. The formation of drug resistance involves many factors, which may be closely related to drug metabolism, autophagy, tumor cell metabolism, signaling pathway and so on. Therefore, understanding the molecular mechanisms of chemoreresistance may provide a basis for the development of therapies for ovarian cancer.

In this paper, we first analyzed the original data of GSE18520, identified 3,852 DEGs, and carried out GO/KEGG enrichment analysis on DEGs. We found that DEGs was correlated with PI3K-AKT and AMPK signaling pathways, and then we took the intersection of DEGs and PI3K-AKT and AMPK related genes to obtain 3 common genes. We found that CRTC2 was associated with ovarian cancer poor prognosis through Kaplan-Meier database. Tissue samples and cell lines were used to investigate the significance of CRTC2 in ovarian cancer. The expression of CRTC2 in ovarian cancer and its corresponding normal tissues was confirmed by immunohistochemical staining in tissue microarray for the first time. The expression of CRTC2 in ovarian cancer was higher than that in the corresponding normal tissues. At the same time, we also discovered that CRTC2 was positively correlated with cancer malignant indicators such as distant metastasis and FIGO stage. Therefore, we studied the effect of CRTC2 on the progress of ovarian cancer cells in SKOV3 and A2780 by CCK-8, clone formation, and transwell assays. These results suggest a potential relationship between CRTC2 and the occurrence and development of ovarian cancer, and its role as a prognostic indicator of ovarian cancer.

CRTC2 is not only highly expressed in a variety of tumors, but also has been proven by a large number of studies that CRTC2 is also closely related to chemotherapy resistance of tumor cells. A clinical trial showed that the combination of CRTC1/CRTC2 inhibitor and lapatinib had a synergistic effect in bladder cancer *in vitro* 27. Sabbah, M et al 28 found that the activation of CRTC2/MITF/Bcl-2 signaling axis could explain the resistance of dasatinib produced by melanoma patients. In the study of invasive and hormone refractory prostate cancer, the inhibitor Palomid 529 of CRTC2 can increase the sensitivity to docetaxel and cisplatin 29. When studying the relationship between CRTC2 and clinical factors, we were surprised to find that patients in the high expression group of CRTC2 were more likely to develop chemotherapy resistance. According to the chemosensitivity test, ovarian cancer cells that inhibit CRTC2 are more sensitive to paclitaxel. On the contrary, cells that overexpress CRTC2 have a slight response to paclitaxel. Based on this, we have every reason to believe that CRTC2 may induce chemoresistance in ovarian cancer cells.

Previous studies have found that PI3K-AKT and AMPK can mediate cell proliferation, differentiation, migration, apoptosis, autophagy and other processes 30. Autophagy is a highly conserved and strictly regulated cellular process, which includes initiation, extension, fusion and lysosomal degradation and maintains the stability of the intracellular environment 31. It can also reduce cell damage by eliminating potential toxic substances and increasing the adaptive capacity of cells 32. Autophagy can enhance the anti-apoptosis ability of tumor cells. When tumor cells are in poor environments such as hypoxia, starvation, infection and physical damage, autophagy is activated and tolerance is enhanced, which helps to maintain cell stability 33. During surgery, radiotherapy and chemotherapy, “induced autophagy” causes therapeutic resistance by promoting the self-dormancy of tumor cells, which is one of the reasons for tumor recurrence and distant metastasis. In this study, the expression of CRTC2 and the level of autophagy in ovarian cancer cells were increased by time and concentration gradient paclitaxel exposure. Subsequently, we found that CRTC2 may affect the chemotherapy sensitivity of ovarian cancer by affecting autophagic flux.

## Conclusion

In summary, we found the expression and function of CRTC2 in ovarian cancer for the first time. In this study, we preliminarily investigated the function and mechanism of CRTC2 by combining molecular information from public databases, clinicopathology and cytology. CRTC2 is highly expressed in ovarian cancer cells and tissues, and the high expression of CRTC2 is associated with the poor prognosis of ovarian cancer patients. Paclitaxel induces the up-regulation of CRTC2 and autophagy level in ovarian cancer cells, thereby promoting the sensitivity of ovarian cancer cells to paclitaxel. However, the specific mechanism of CRTC2 affecting autophagy needs to be further studied.

## Figures and Tables

**Table 1:** The association of CRTC2 expression with clinicopathological parameters in ovarian cancer

Characteristics	Group	CRTC2		χ² value	P value
		Low-exp	High-exp		
Age	≤60	35	36	0.329	0.566
	>60	17	22		
Pausimenia	No	12	12	0.092	0.760
	Yes	40	46		
Distant metastasis	No	30	18	7.922	0.005**
	Yes	22	40		
Neoadjuvant chemotherapy	No	25	17	4.091	0.043*
	Yes	27	41		
Ascites	No	28	22	2.801	0.094
	Yes	24	36		
FIGO stage	Ⅰ-Ⅱ	17	8	5.576	0.018*
	Ⅲ-Ⅳ	35	50		
Lymphatic metastasis	No	32	25	3.732	0.053
	Yes	20	33		
Chemosensitivity	No	20	45	6.758	0.009**
	Yes	25	20		

Annotations: *p<0.05, **p<0.01.
